# Supplementary material for: Implementation of residue-level coarse-grained models in GENESIS for large-scale molecular dynamics simulations
Source: PLoS Comput Biol. 2022 Apr 5;18(4):e1009578. doi: 10.1371/journal.pcbi.1009578 (PMC9012402; doi:10.1371/journal.pcbi.1009578)
Supplement: S1 Text — (DOCX) [file pcbi.1009578.s001.docx]

**Supporting Information on**

**Implementation of residue-level coarse-grained models in GENESIS for large-scale molecular dynamics simulations**

Cheng Tan^1^, Jaewoon Jung^1,2^, Chigusa Kobayashi^1^, Diego Ugarte La Torre^1^, Shoji Takada^3^, Yuji Sugita^1,2,4,*^

^1^ Computational Biophysics Research Team, RIKEN Center for Computational Science, Kobe, Hyogo, Japan

^2^ Theoretical Molecular Science Laboratory, RIKEN Cluster for Pioneering Research, Wako, Saitama, Japan

^3^ Department of Biophysics, Graduate School of Science, Kyoto University, Kyoto, Japan

^4^ Laboratory for Biomolecular Function Simulation, RIKEN Center for Biosystems Dynamics Research, Kobe, Hyogo, Japan

^*^ [sugita@riken.jp](mailto:sugita@riken.jp)

**Supplementary Methods**

**Sry target search on DNA.** We employed the AICG2+ model [1] for protein ($V_{AICG2+}$) and the 3SPN.2C model [2,3] for DNA ($V_{3SPN.2C}$). For protein-DNA sequence-nonspecific binding, we considered Debye-Hückel electrostatic interactions ($E_{ele}$) and excluded volume interactions ($E_{exv}^{(2)}$). As for the sequence-specific interactions between sry and DNA bases, we used the PWMcos model ($E_{PWMcos}$) [4], with parameters $\epsilon^{'}=-0.1 kcal/mol$ and $\gamma=2.8$, which were taken from recent study of another HMG protein, Sox2 [5]. Note that for a more accurate modeling, these parameters should be recalibrated by comparing with experimental results of sry-DNA binding. All the other parameters were using the default values of the original models.

**FUS IDR condensation.** The amino-acid sequence of the FUS IDR used in our simulations was: MASNDYTQQATQSYGAYPTQPGQGYSQQSSQPYGQQSYSGYSQSTDTSGYGQSSYSSYGQSQNTGYGTQSTPQGYGSTGGYGSSQSSQSSYGQQSSYPGYGQQPAPSSTSGSYGSSSQSSSYGQPQSGSYSQQPSYGGQQQSYGQQQSYNPPQGYGQQNQYNS (163aa).

**Native-ness coordinate of protein folding.** In the current work, we define the native-ness of a protein conformation as: $Q(\Gamma)=n_{nat}(\Gamma)/N_{nat}$, where $N_{nat}$ is the total number of native contacts, and $n_{nat}(\Gamma)$ is the number of formed native contacts in a given structure $\Gamma$. When a native-contact pair of particles is within 1.2 times of their native distance, the contact is considered to be formed, otherwise not formed.

**Modeling of the artificial chromatin.** To construct the artificial chromatin, we first built the di-nucleosome structures by connecting two nucleosomes (using PDB 1KX5 as a template) with linker DNAs. The di-nucleosomes were then put in a 3-D grid connected by an order-3 Hilbert filling curve. More longer DNAs were used between the neighboring nodes, and all the DNA strands were connected by rotating the di-nucleosomes and linker DNAs to minimize the DNA end distances. In the final structure there is only one dsDNA, with the sequence of poly-CG. The constructed structure was first energy-minimized for 5500 steps using the steepest descent algorithm and then equilibrated for ${10}^{5}$ steps at 300K.

**Histone tail indices.** For the histones, we used the AICG2+ model [1] for the folded domains and the HPS model [6] for the histone tails. The indices of the tails are: 1-32 in H3, 1-23 in H4, 1-14 (N-terminal) and 121-128 (C-terminal) in H2A, and 1-26 in H2B. For Fig 8C, only the N-terminal tails were used for analysis for each histone.

**Reference**

1. Li W, Wang W, Takada S. Energy landscape views for interplays among folding, binding, and allostery of calmodulin domains. Proc Natl Acad Sci. 2014;111: 10550–10555. doi:10.1073/pnas.1402768111

2. Hinckley DM, Freeman GS, Whitmer JK, de Pablo JJ. An experimentally-informed coarse-grained 3-site-per-nucleotide model of DNA: Structure, thermodynamics, and dynamics of hybridization. J Chem Phys. 2013;139: 144903. doi:10.1063/1.4822042

3. Freeman GS, Hinckley DM, Lequieu JP, Whitmer JK, de Pablo JJ. Coarse-grained modeling of DNA curvature. J Chem Phys. 2014;141: 165103. doi:10.1063/1.4897649

4. Tan C, Takada S. Dynamic and Structural Modeling of the Specificity in Protein–DNA Interactions Guided by Binding Assay and Structure Data. J Chem Theory Comput. 2018;14: 3877–3889. doi:10.1021/acs.jctc.8b00299

5. Tan C, Takada S. Nucleosome allostery in pioneer transcription factor binding. Proc Natl Acad Sci. 2020;117: 20586–20596. doi:10.1073/pnas.2005500117

6. Dignon GL, Zheng W, Kim YC, Best RB, Mittal J. Sequence determinants of protein phase behavior from a coarse-grained model. PLOS Comput Biol. 2018;14: e1005941. doi:10.1371/journal.pcbi.1005941
