# Supplementary figures and images for: Implementation of residue-level coarse-grained models in GENESIS for large-scale molecular dynamics simulations
Source: PLoS Comput Biol. 2022 Apr 5;18(4):e1009578. doi: 10.1371/journal.pcbi.1009578 (PMC9012402; doi:10.1371/journal.pcbi.1009578)

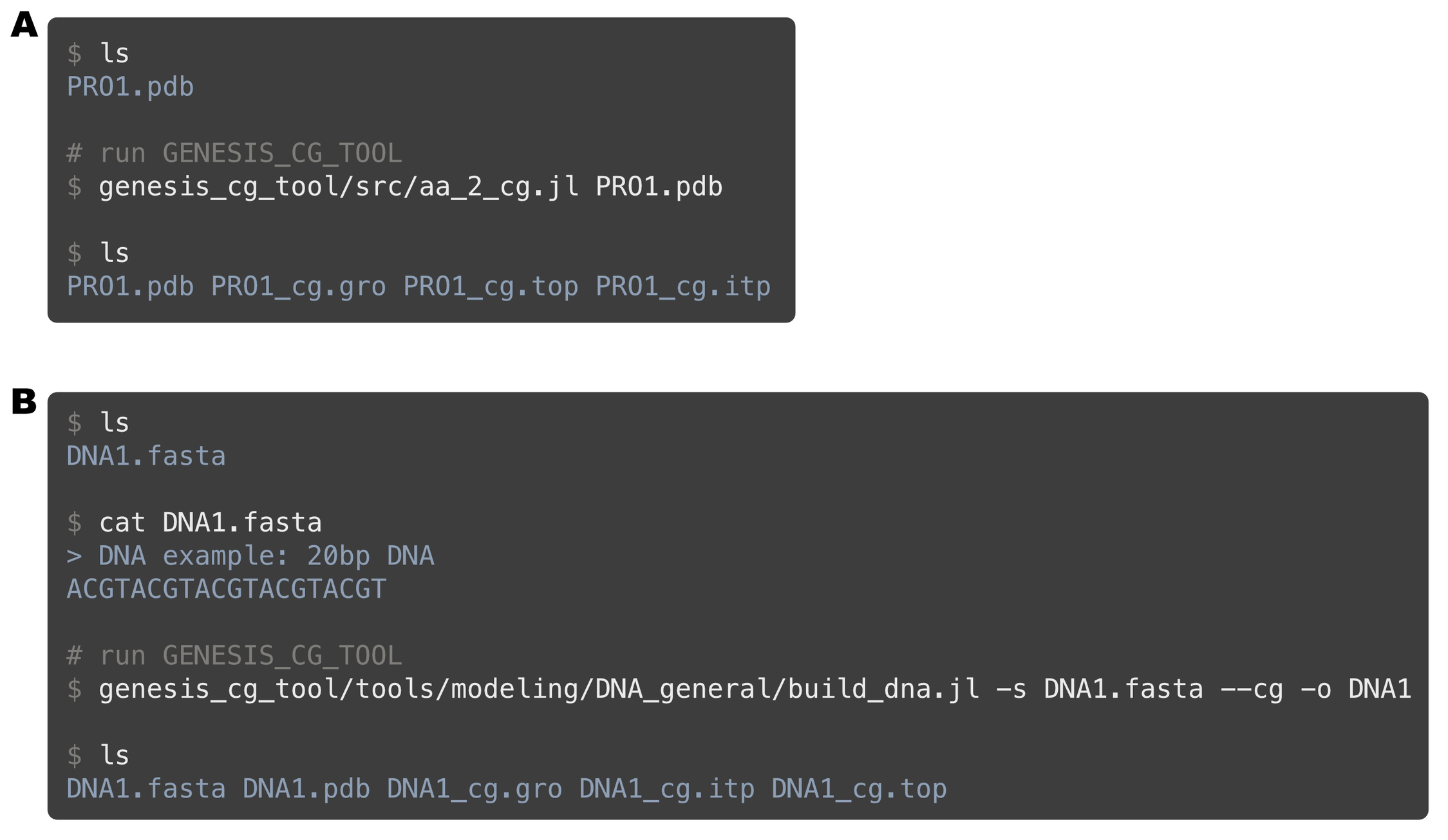

Supplement: S1 Fig — (A) Generate CG files (PRO1_cg.gro for coordinates; PRO1_cg.top and PRO1_cg.itp for topology) for a protein, based on its atomistic PDB file (PRO1.pdb). (B) Generate CG files (DNA1_cg.gro for coordinates; DNA1_cg.top and DNA1_cg.itp for topology) as well as an atomistic coordinate file (DNA1.pdb) for a 20bp DNA, from its DNA sequence (DNA1.fasta). (TIF) [file pcbi.1009578.s002.tif]

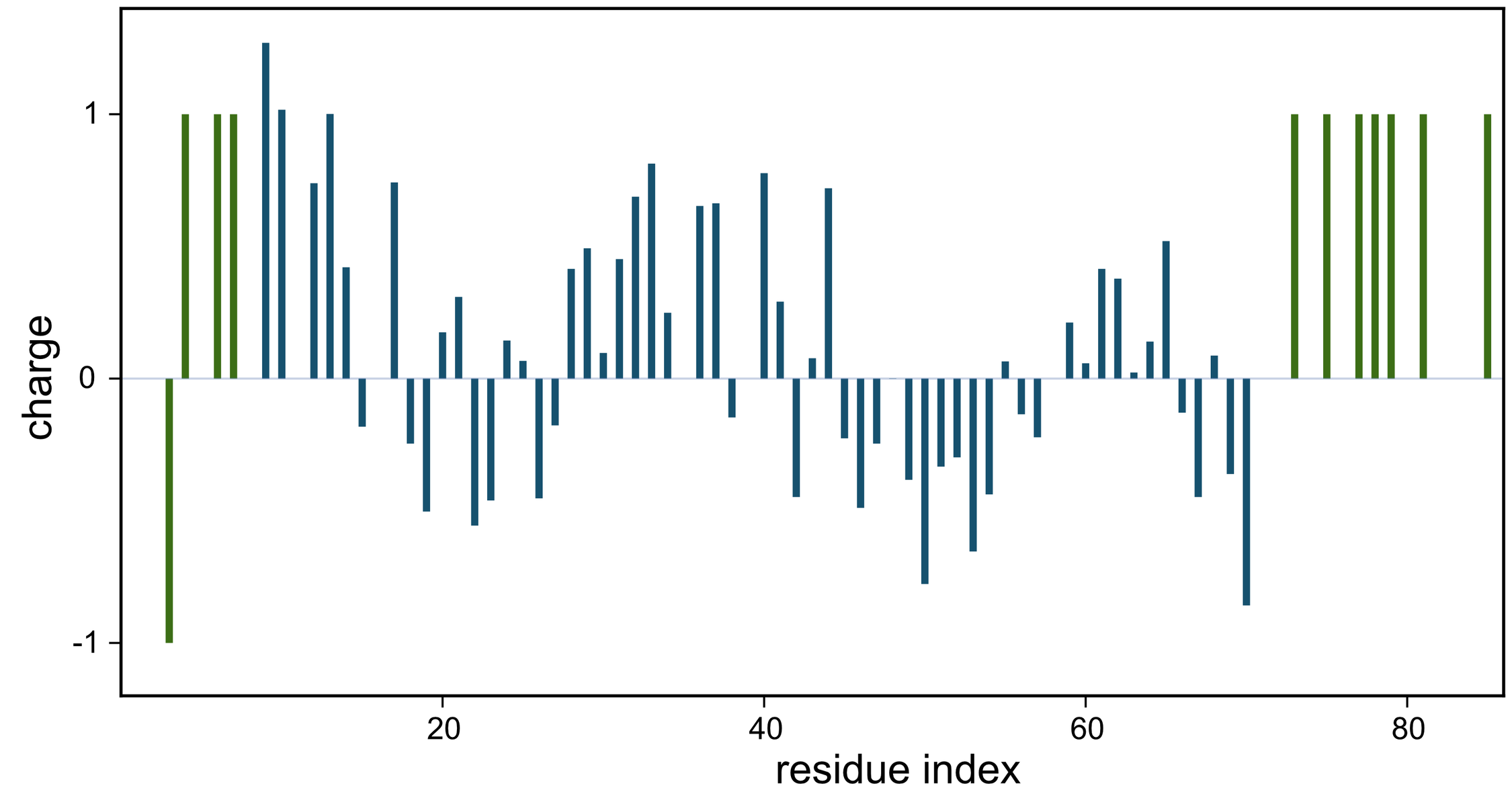

Supplement: S2 Fig — The charges of the surface residues of the HMG domain (residue 9–70, dark blue) of sry was determined with the RESPAC method. Whereas the charges on the N-tail and C-tail were the original integer charges (green). The same data are used to plot the CG structure of sry (Fig 5B). (TIF) [file pcbi.1009578.s003.tif]
